# The analysis of gut microbiota characteristics in children with global developmental delay

**DOI:** 10.3389/fcimb.2025.1606453

**Published:** 2026-01-05

**Authors:** Lingyu Wan, Congfu Huang, Wei Kong, Meifen Li, Chengyu Lu

**Affiliations:** 1 Department of Pediatrics, Longgang District Maternity & Child Healthcare Hospital of Shenzhen City (Affiliated Shenzhen Women and Children's Hospital (Longgang) of Shantou University Medical College), Shenzhen, China; 2 Department of Pediatrics, The First Affiliated Hospital of Guangzhou Medical University, Guangzhou, China

**Keywords:** 16S rDNA, characteristics, global developmental delay (GDD), gut microbiota, high-throughput sequencing

## Abstract

**Objective:**

To explore the composition and functional changes of gut microbiota in children with Global Developmental Delay(GDD),and to explore the role of gut microbiota in the pathogenesis of GDD using high-throughput sequencing.

**Methods:**

A prospective study was conducted to select 26 children diagnosed with GDD at Longgang District Maternal and Child HealthCare Hospital of Shenzhen City from January 2024 to December 2024 as the disease group(GDD), and 59 healthy children of the same age were selected as the healthy group(HC).General information of the children was collected through a questionnaire survey, and fecal samples from all participants were collected. Total DNA was extracted and amplified, and high-throughput sequencing of the 16S rRNA gene was performed for biological analysis of the sequencing results.

**Results:**

The alpha diversity analysis revealed a significant reduction in microbial diversity in the GDD group (Chao1 index, P = 0.007), while the beta diversity showed significant segregation between groups (R² = 0.067, P = 0.001);At the phylum level, the relative abundance of Actinobacteria was significantly increased (P < 0.01), while the abundance of Bacteroidetes was significantly decreased (P < 0.05) in the GDD group;At the genus level, the abundance of Bifidobacterium, Fusicatenibacter, and Erysipelatoclostridium were significantly increased in the GDD group (all P < 0.001), while the abundance of Faecalibacterium, Phascolarctobacterium, and Alistipes were significantly reduced (all P < 0.001);Functional prediction based on 16S rRNA data suggested potential differences in microbial metabolic pathways, including mRNA surveillance, proteasome, and atrazine degradation, in the GDD group. These findings hypothesize a functional shift in the gut microbiome associated with GDD, which requires validation by direct metagenomic or metabolomic methods.

**Conclusion:**

Children with GDD have significant differences in gut microbiota composition and diversity compared to HC,and the abundance and abnormal metabolic pathway may be closely related to the neuroinflammatory process, suggesting that intestinal microecological regulation may become a new intervention target for GDD.

## 1 Introduction

Global Developmental Delay (GDD) is a neurodevelopmental disorder characterized by significant delays across multiple developmental domains—including cognition, language, motor skills, daily living skills, and social abilities—compared to age-expected milestones (Vickers and Gibson, 2019). It has a global prevalence of approximately 1–3% (Miclea et al., 2015). Currently, the diagnosis of GDD primarily relies on behavioral assessments and neuroimaging techniques, yet there remains a lack of objective biomarkers for this condition (Song et al., 2024). To date, the underlying pathogenesis of GDD has not been fully elucidated (Volpedo et al., 2025). With growing interest in the microbiota–gut–brain axis, the gut microbiota has emerged as a critical environmental factor influencing neurodevelopment (Tognini, 2017). Accumulating clinical evidence suggests that gut dysbiosis is closely associated with the pathophysiology of various neurodevelopmental disorders in children. For instance, children with Cerebral Palsy (CP) commonly exhibit reduced gut microbial α-diversity and an increased relative abundance of opportunistic pathogens such as *Enterobacteriaceae* (Peng et al., 2023). Such microbial imbalance may exacerbate motor impairments by triggering systemic low-grade inflammation. Similarly, patients with Attention Deficit Hyperactivity Disorder (ADHD) show marked reductions in beneficial bacteria like *Lactobacillus* and *Bifidobacterium* (Wang et al., 2025). These alterations in the gut microbiota may contribute to disruptions in dopaminergic signaling via interference with the tryptophan–dopamine metabolic pathway. Recent clinical studies further highlight the role of bidirectional communication along the microbiota–gut–brain axis, mediated through metabolic, endocrine, neural, and immune pathways, thereby providing a theoretical foundation for investigating how gut microbiota influences GDD (Cryan and Dinan, 2012). However, while numerous studies have explored gut microbiota profiles in autism spectrum disorder (ASD), CP, and ADHD, research focusing on GDD remains limited, particularly regarding the functional mechanisms of microbial communities in this disorder.

To address this gap, the present study employed 16S rRNA sequencing to analyze and compare the abundance, composition, species distribution, and functional prediction of gut microbiota between children diagnosed with GDD and age-matched healthy controls (HC). By examining differences in microbial diversity and abundance between the two groups, this study aims to identify potential gut microbial biomarkers for GDD. The findings are expected to provide an objective basis for early and accurate diagnosis, as well as potential targets for therapeutic intervention in children with global developmental delay.

## 2 Materials and methods

### 2.1 Sample selection

This study employed a convenience sampling method from January 2024 to December 2024, selecting 26 children diagnosed with GDD attending the Longgang District Maternal and Child HealthCare Hospital of Shenzhen City as the disease group(GDD). Simultaneously, 59 age-matched healthy children who came for routine check-ups at the hospital were selected as the healthy group(HC). Inclusion criteria: age ≤ 5 years; meeting the diagnostic criteria for GDD, as defined by a developmental quotient (DQ) of < 70 in at least two domains of the Gesell Developmental Schedules (GDS) (National Center for Children's Health, et al., 2024); guardians provided informed consent and were willing to cooperate. Exclusion criteria: ①chronic gastrointestinal diseases such as inflammatory bowel disease and ulcerative colitis; ②severe liver and kidney diseases; ③use of antibiotics or probiotics within the 4 weeks prior to enrollment. This study was approved by the hospital’s Ethics Committee (Ethics Approval No: LGFYKYXMLL-2024-103), and informed consent was obtained from all guardians.

### 2.2 Sample size calculation

This study adopts a sample size calculation method based on effect size, and the detailed calculation formula is as follows (Equation 1). The mean value of Chao1 in the HC group is 200(SD = 50), and the mean value of the GDD group is 150(SD = 50). Therefore, Δ=50, σ=50. By substituting these values, it is calculated that each group requires at least 17 cases(with a total sample size of 34 cases). This study selected 26 children with comprehensive developmental delay as the disease and 59 healthy children of the same age who underwent physical examination in the pediatric health department as the healthy group. The final sample size of 26 GDD and 59 HC participants exceeded the minimum requirement of 17 per group, thus satisfying the statistical power criteria.

(1) $$n=\;\frac{2\;\times \;{\left(Z_{\alpha /2}\;+\;Z_{\beta }\right)}^{2}\;\times \;\sigma^{2}}{\Delta^{2}}$$

The sample size was calculated using the following formula for comparing two means:

https://media/image2.jpeg{width=“2.6in” height=“0.7666666666666667in”}.

Where:

n is the required sample size per group.

Zα is the Z-value for the Type I error rate (α); we set α = 0.05 (two-tailed), hence Zα = 1.96.

Zβ is the Z-value for the Type II error rate (β) or statistical power (1-β); we set β = 0.20 (i.e., 80% power), hence Zβ = 0.84.

Δ is the expected mean difference in the Chao1 index between groups (Δ = 50).

σ is the common standard deviation (σ = 50).

### 2.3 Research methods

#### 2.2.1 Collection of baseline data

Design a survey questionnaire according to the research objectives,which includes basic information such as children’s age, BMI value, gender, delivery mode, family residence,feeding type and dietary patterns etc.

#### 2.2.2 Fecal sample collection and storage

Fecal samples (3-5g) were collected from both groups of children using sterile collection tubes. Immediately after collection, the samples were transported to a -80 °C freezer for storage and later sent to Shenzhen Huada Precision Nutrition Technology Co., Ltd. for high-throughput sequencing analysis.

#### 2.2.3 DNA extraction, library preparation, and sequencing

The PowerSoil^®^ DNA Isolation Kit (MoBio) was used to extract total bacterial DNA from the fecal samples. The extracted DNA was then subjected to PCR amplification of the V3-V4 region of the 16S rRNA gene. The amplified sequences were sequenced using the Illumina Miseq high-throughput sequencing platform.

#### 2.2.4 Bioinformatics analysis

The sequencing data were processed through self-developed bioinformatics tools for quality filtering, followed by sequence assembly using FLASH software (v1.2.11, http://ccb.jhu.edu/software/FLASH/index.shtml). The assembled sequences were clustered into OTUs (Operational Taxonomic Units) using USEARCH. Data analysis was primarily conducted using Qiime2 and R software version 3.2.0. The α-diversity index (Simpson) was calculated using Qiime2 to evaluate species richness and evenness. Principal Component Analysis (PCA) based on unweighted UniFrac distance was performed to assess inter-group differences in species composition. The bacterial composition of all samples was annotated using the Greengene V201305 database. Differential abundance of bacteria at the phylum, class, order, family, and genus levels between the GDD and HC was assessed.

### 2.4 Statistical methods

The baseline data of children were processed using SPSS 26.0 software. Count data was described using frequency, rate, and composition ratio, and between group comparisons were performed using chisquare test, continuous variables that conformed to a normal distribution are presented as mean ± standard deviation. Differences in continuous baseline characteristics (e.g., age, BMI) between the two groups (GDD vs. HC) were assessed using an independent samples t-test. Differences in alpha diversity indices (e.g., Chao1) between the two groups (GDD vs. HC) were assessed using independent samples t-tests. For beta diversity, permutational multivariate analysis of variance (PERMANOVA) was applied based on unweighted UniFrac distances. If the ANOVA result was significant (P < 0.05), *post-hoc* pairwise comparisons were conducted using Tukey’s Honest Significant Difference (HSD) test to identify which specific groups differed. Principal component analysis (PCA) was performed using the ade4 package in R (v3.3.3) based on the composition and relative abundance of bacterial genera. The overall bacterial distribution of the two groups was visualized. The Wilcoxon method was used to test for differential species between the two groups at the Phylum, Genus, and Species levels, with *P*<0.05 considered statistically significant. Functionality of the gut microbiome was inferred using KEGG (Kyoto Encyclopedia of Genes and Genomes) database, and functional differences between the two groups were analyzed.

## 3 Results

### 3.1 Comparison of baseline data between the two groups

There was no statistically significant differences in the demographic characteristics and disease-related information between the two groups (*P*>0.05), indicating age, BMI value, gender,delivery mode, family residence, feeding type and dietary patterns etc (**Table 1**). (Note: Although detailed dietary records were not collected, the use of nutritional supplements (e.g., vitamins, minerals) between the GDD and HC groups showed no statistically significant difference (P = 0.638, **Table 1**). This suggests that gross differences in supplement intake are unlikely to be the primary driver of the observed microbiota disparities, though the influence of unmeasured specific dietary components cannot be ruled out.).

**Table 1:** Comparison of baseline data between two groups of children.

Variables	GDD(n=26)	HC(n=59)	Statistic	*P*
Age(years)	3.00 ± 1.30	3.03 ± 1.29	0.112	0.911
BMI(Kg/m^2^)	21.31 ± 1.87	22.51 ± 0.85	0.034	0.830
Gender			χ²=0.696	0.404
Male	19(73.1%)	37(62.7%)		
Female	7(26.9%)	22(37.3%)		
Delivery mode			1.539	0.226
Caesarean section	20(76.9%)	49(83.1%)		
Natural childbirth	6(23.1%)	10(16.9%)		
Family residence			0.056	0.840
Rural area	12(46.2%)	39(66.1%)		
Town	14(53.8%)	20(33.9%)		
Feeding type			0.065	0.799
Breast feeding	14(53.8%)	30(50.8%)		
Artificial feeding	12(46.2%)	29(49.2%)		
Dietary supplement			0.222	0.638
Use	12(46.2%)	24(40.7%)		
Not use	14(53.8%)	35(59.3%)		

Categorical variables are presented as numbers (percentages) [n (%)], and comparisons between groups were performed using the Chi square test. Continuous variables that conform to a normal distribution are represented as mean ± standard deviation (Mean ± SD), and inter group comparisons are conducted using independent sample t-test (Continuous variables conforming to a normal distribution are presented as mean ± standard deviation (Mean ± SD)) SD), and inter-group comparisons were analyzed using the independent samples t-test)。 P<0.05 is considered statistically significant. The use of dietary supplements “is defined as any commercial nutritional supplements beyond normal diet, such as multivitamins, vitamin D, calcium, iron, or omega-3 fatty acids, that are currently regularly consumed outside of normal diet, as reported by the guardians. GDD, Global Developmental Delay; HC, Healthy control group.

### 3.2 Comparison of gut microbiota diversity

**Figure 1:**
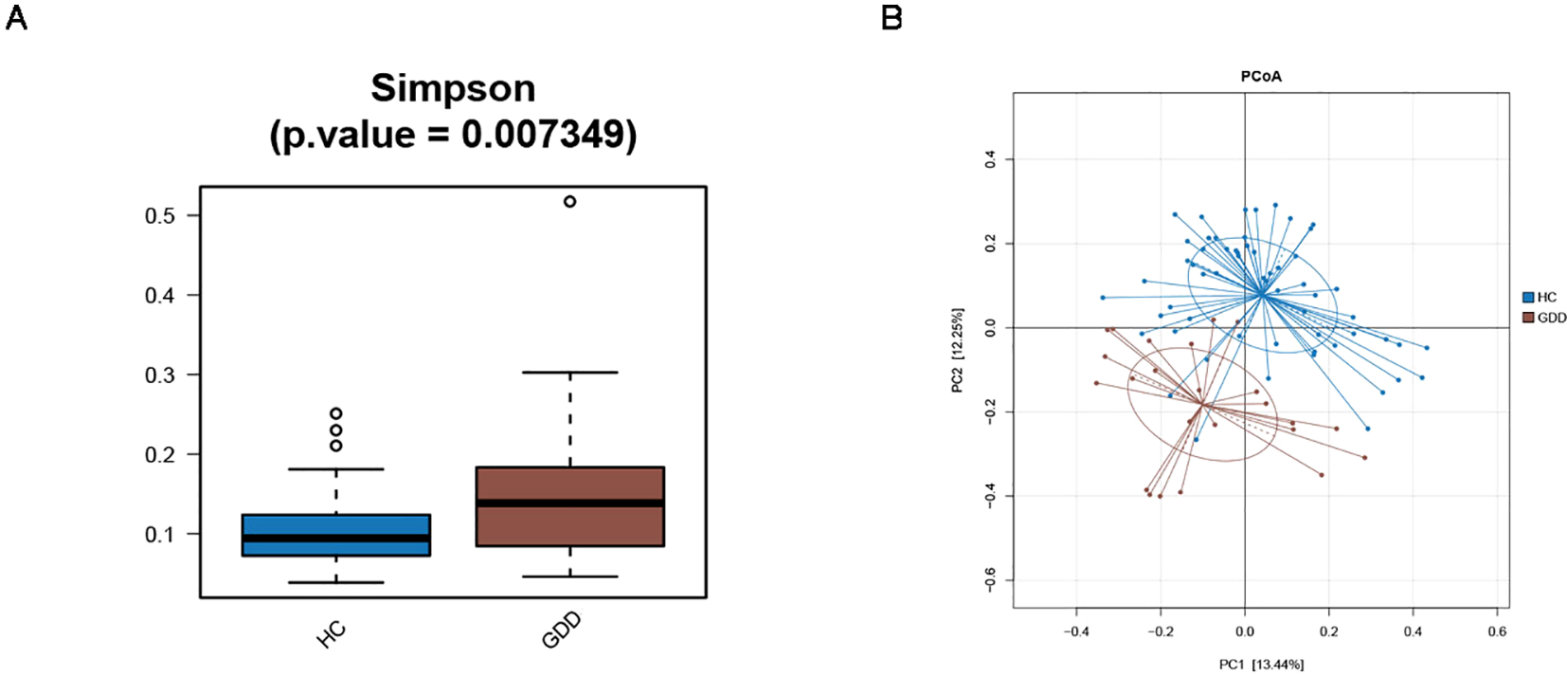
Comparison of gut microbiota diversity between GDD and HC groups. **(A)** Alpha diversity as measured by the Simpson. Data are presented as box plots. The difference between groups was assessed by independent samples t-test (P = 0.007). **(B)** Beta diversity visualized by Principal Coordinate Analysis (PCoA) based on unweighted UniFrac distances. The variance explained by each principal coordinate is indicated in parentheses. Group separation was tested using PERMANOVA (R² = 0.067, *P* = 0.001). GDD, Global Developmental Delay (n=26); HC, Healthy Control (n=59).

The analysis results indicated that: 1) An independent samples t-test revealed a significant difference in microbial alpha diversity (Chao1 index) between the two groups of children (P = 0.007) (**Figure 1A**). 2) Principal coordinate analysis (PCoA) was used to perform dimensionality reduction on the gut microbiota data of both groups. The β-diversity of gut microbiota composition showed a clear separation between the two groups (**Figure 1B**).

### 3.3 Analysis of dominant phyla and differences between the two groups

**Figure 2:**
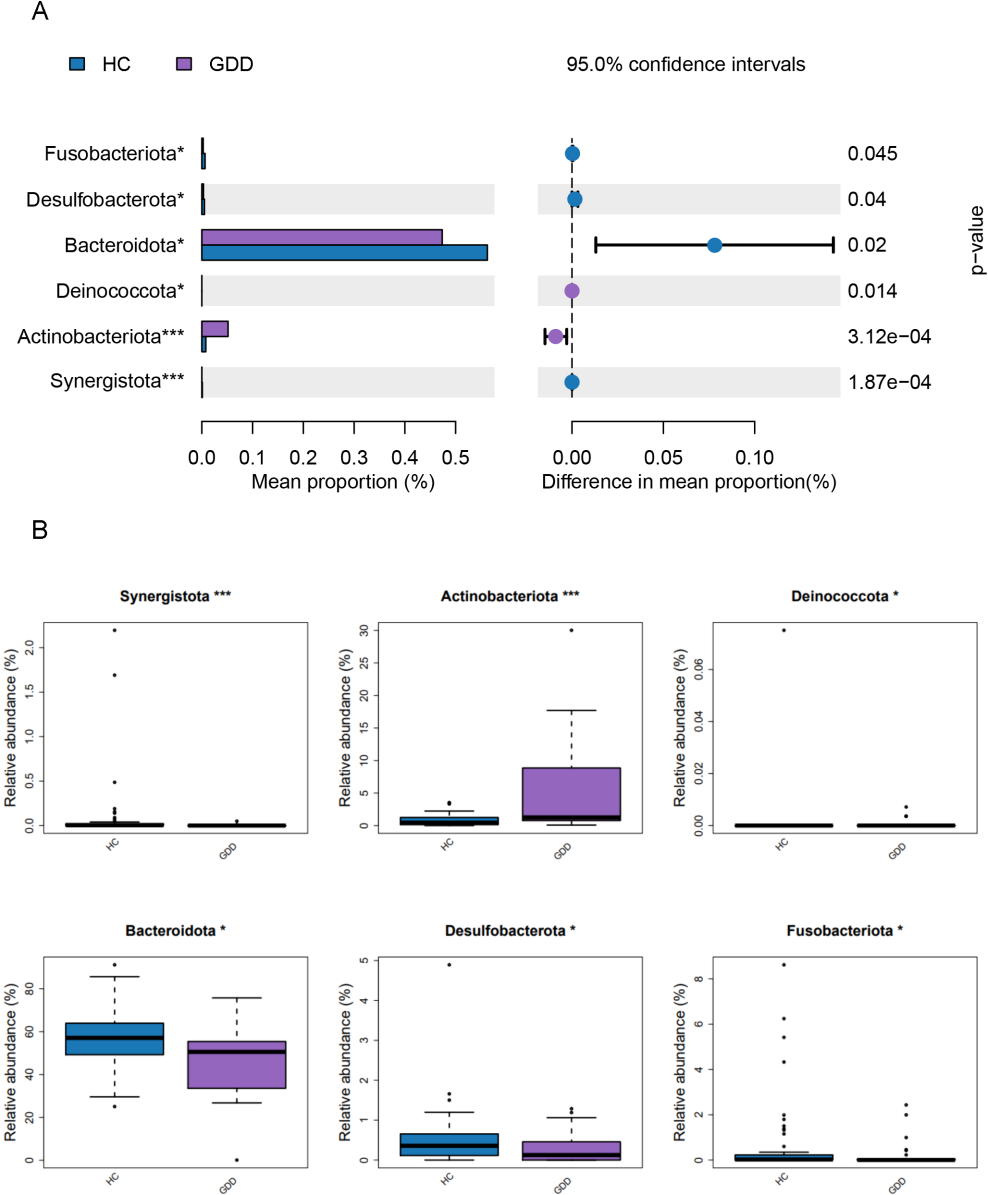
Composition and differential abundance of dominant bacterial phyla in GDD and HC groups.**(A)** Histogram of the LDA Effect Size (LEfSe) analysis, showing the taxonomicphyla that were significantly enriched in either the GDD or HC group. The histogram displays the Linear Discriminant Analysis (LDA) score (log10) for each discriminative feature. Genera are ranked by their LDA score, with the length of the bar representing the effect size. The color of the bars indicates the group in which the genus is enriched (violet: GDD; blue: HC). **(B)** Box plot illustrating the relative abundance of the two phyla that showed statistically significant differences between groups: *Actinobacteria* and *Bacteroidetes*. The boxes represent the interquartile range (IQR), the horizontal line inside the box indicates the median, and whiskers extend to 1.5×IQR. Individual data points are overlaid.Differential abundance at the phylum level was tested using the Wilcoxon rank-sum test; *P < 0.05 and ***P < 0.001 indicate statistical significance.GDD, Global Developmental Delay (n=26); HC, Healthy Control (n=59).

The analysis of dominant bacterial phyla in both groups is shown in **Figure 2**. At the phylum level, the relative abundance of *Actinobacteria* was significantly higher in the GDD compared to the HC (*P*< 0.01), while *Bacteroidetes* was significantly lower in the GDD than in the HC(*P*< 0.05).

### 3.4 Analysis of dominant genus and differences between the two groups

**Figure 3:**
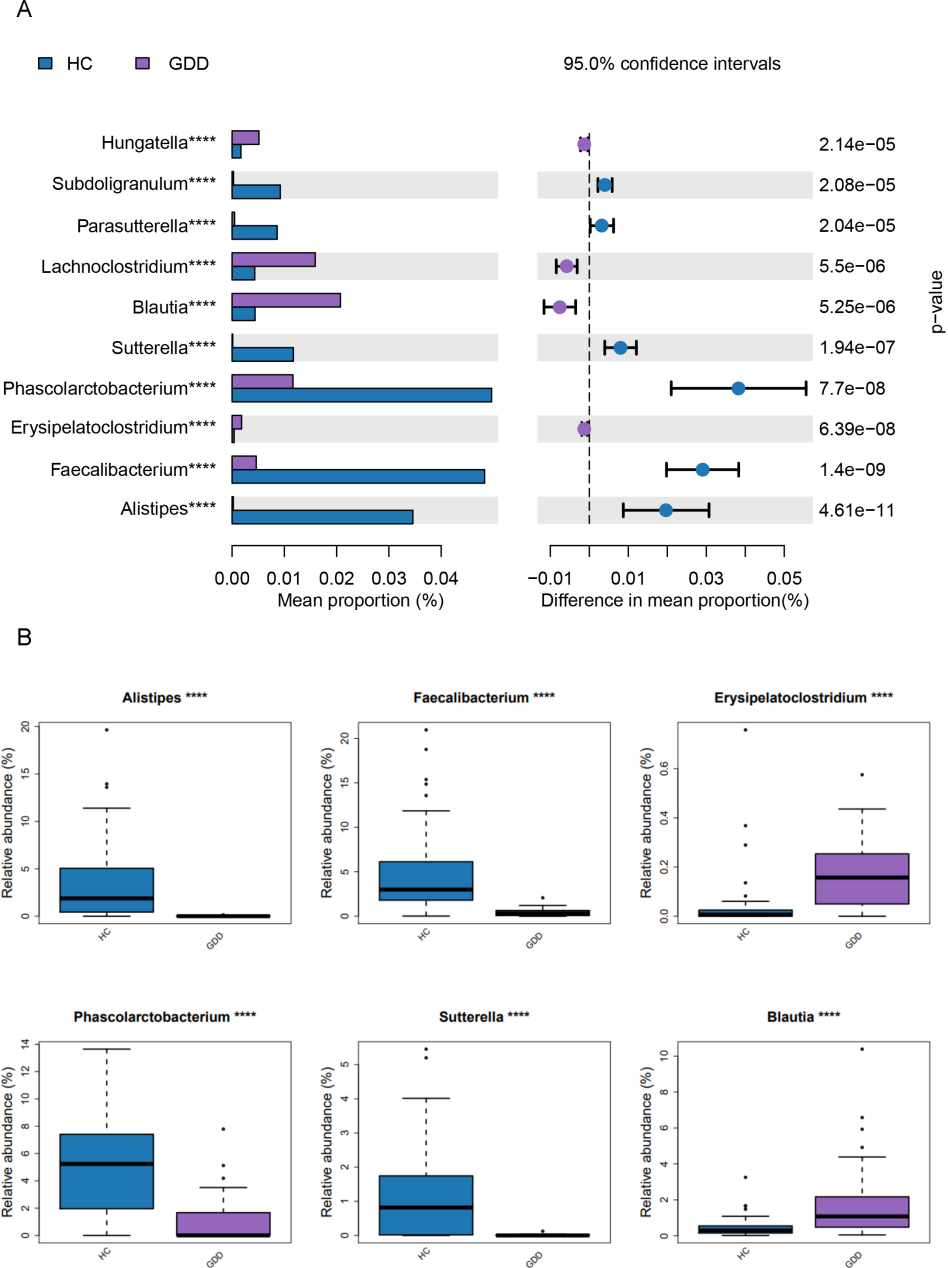
Differential abundance analysis of gut microbiota at the genus level between GDD and HC groups. **(A)** Histogram of the LDA Effect Size (LEfSe) analysis, showing the taxonomic genera that were significantly enriched in either the GDD or HC group. The histogram displays the Linear Discriminant Analysis (LDA) score (log10) for each discriminative feature. Genera are ranked by their LDA score, with the length of the bar representing the effect size. The color of the bars indicates the group in which the genus is enriched (violet: GDD; blue: HC). **(B)** Box plots comparing the relative abundance (%) of four selected genera that were significantly depleted in the GDD group compared to the HC group: *Faecalibacterium*, *Phascolarctobacterium*, *Alistipes*, and *Sutterella*. The boxes represent the interquartile range (IQR), the horizontal line inside the box indicates the median, and the whiskers extend to 1.5×IQR. Individual data points are overlaid. The asterisks ( * ) denote the level of statistical significance ( ****P < 0.0001) as determined by the Wilcoxon rank-sum test.GDD, Global Developmental Delay (n=26); HC, Healthy Control (n=59).

At the genus level, we selected the top six dominant bacterial genera in both groups and calculated their relative abundances (**Figures 3**). The analysis results revealed the following:Genera significantly more abundant in the GDD compared to the HC: *Lachnoclostridium, Blautia,Hungatella* and *Erysipelatoclostridium (all P< 0.00*1).Genera significantly less abundant in the GDD compared to the HC: *Faecalibacterium, Phascolarctobacterium, Alistipes* and *Sutterella* (all *P* < 0.001).

### 3.5 Functional characteristics of gut microbiota

**Figure 4:**
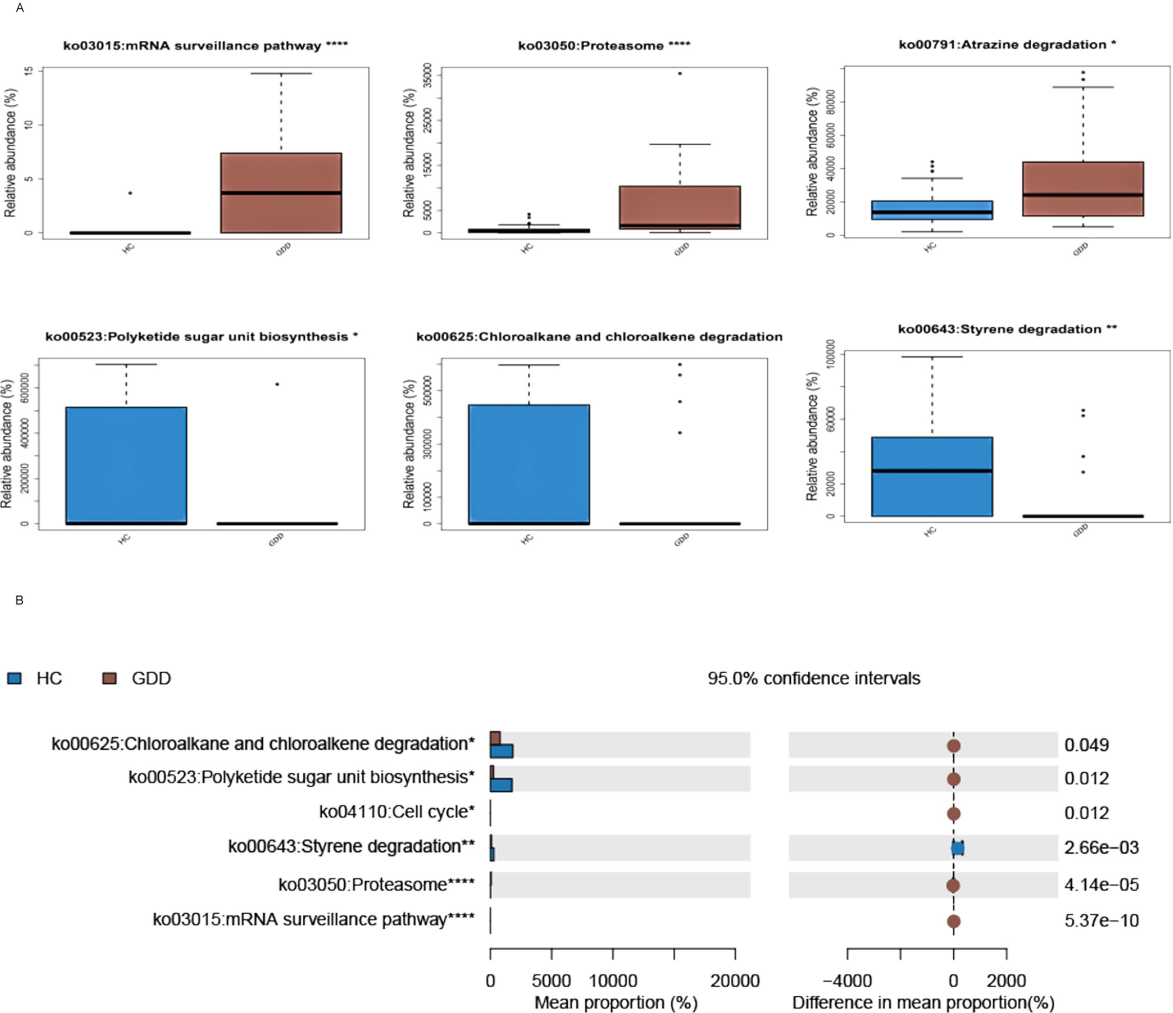
Predicted functional alterations of the gut microbiome in GDD children based on KEGG pathway analysis. **(A)** Bar plot of the Linear Discriminant Analysis (LDA) Effect Size (LEfSe) showing KEGG pathways that were significantly enriched in either the GDD or HC group. The length of each bar represents the LDA score (log10), indicating the effect size of each differentially abundant pathway. Pathways are ranked by their LDA score. The color of the bars indicates the group in which the pathway is enriched (violet: GDD; blue: HC). Only pathways with an LDA score > 2.0 and a P value < 0.05 (Wilcoxon rank-sum test) are displayed. **(B)** Box plots illustrating the predicted relative abundances (based on PICRUSt2) of six representative KEGG pathways that showed the most significant differences between groups: mRNA surveillance pathway, Proteasome, Atrazine degradation, Polyketide sugar unit biosynthesis, Chloroalkane and chloroalkene degradation, and Styrene degradation. The boxes represent the interquartile range (IQR), the horizontal line inside the box indicates the median, and whiskers extend to 1.5×IQR. Individual data points are overlaid. Statistical significance was determined by the Wilcoxon rank-sum test; *P < 0.05, **P < 0.01, ***P < 0.001, ****P < 0.0001. All functional predictions were inferred from 16S rRNA sequencing data using PICRUSt2. GDD, Global Developmental Delay (n=26); HC, Healthy Control (n=59).

Compared with the HC, functional prediction based on 16S rRNA data suggested potential upregulation of gut microbiota functions in the GDD group, including mRNA surveillance pathway, Proteasome pathway, and Atrazine degradation pathway. Conversely, significantly downregulated functions included Polyketide sugar unit biosynthesis, Chloroalkane and chloroalkene degradation, and Styrene degradation (**Figures 4**).

## 4.Discussion

### 4.1 Research background

The microbiota–gut–brain axis has emerged as a key focus in understanding neurodevelopmental disorders, with growing evidence supporting its role in modulating brain function and behavior via immune, metabolic, and neural pathways (Cryan and Dinan, 2012). Alterations in gut microbial composition have been consistently reported in conditions such as ASD, CP, and ADHD. For instance, individuals with ASD often exhibit a reduced ratio of *Bacteroidetes* to *Firmicutes*, decreased *Prevotella*, and overgrowth of *Clostridium*—patterns that correlate with behavioral symptoms (Ye et al., 2021). Similarly, children with CP demonstrate diminished microbial diversity and increased pathogenic bacteria, which may contribute to motor deficits and chronic inflammation (Peng et al., 2023). In ADHD, reduced levels of *Lactobacillus* and *Bifidobacterium* have been implicated in disrupted dopamine metabolism, potentially exacerbating core symptoms (Wan et al., 2020). Despite these advances, the gut microbiota profile of children with GDD—a common early manifestation of various neurodevelopmental disorders—remains poorly characterized. GDD often represents a prodromal or transitional stage toward more specific diagnoses such as ASD, suggesting that early microbial disturbances may play a formative role in disease progression. Supporting this, microbiota-directed interventions in ASD, including probiotic supplementation and fecal microbiota transplantation, have shown potential to alleviate certain neurobehavioral abnormalities (Zhang et al., 2022), underscoring the therapeutic relevance of targeting the gut–brain axis during early development.

### 4.2 Characteristic differences in the gut microbiota of GDD and HC groups

This study systematically compared the characteristics of the gut microbiota between children in the GDD group and the HC group. First, it was found that there was a significant difference in the overall community structure of gut microbiota between children with GDD and the HC group (*P*<0.01), which was specifically manifested as a change in α diversity. This finding is consistent with the study by Fu et al. (Fu et al, 2025), mainly because the abnormal development of the central nervous system in children with GDD can affect intestinal motility, secretory function, and intestinal barrier integrity through pathways such as the autonomic nerve and the hypothalamic-pituitary-adrenal axis (Lou et al., 2022). Second, at the classification level, the gut microbiota of children with GDD showed obvious loss of balance at both the phylum level and the genus level. At the phylum level, the relative abundance of *Actinobacteria* in the GDD group was significantly increased (*P*<0.01), while the relative abundance of *Bacteroidetes* was significantly decreased (*P*<0.05).This finding contrasts with the conclusion of a study by Borghi et al. on children with CDKL5 deficiency disorder (Borghi et al., 2024). Children with GDD have a monotonous diet or nutritional malabsorption, which may inhibit the colonization of *Bacteroidetes* (Nistal et al., 2012). In addition, GDD studies may include mucosal biopsy samples, which are more likely to test for mucosa-associated *Bacteroidetes*.

At the genus level, the GDD group exhibited significantly higher relative abundances of *Lachnoclostridium, Blautia, Hungatella*, and *Erysipelatoclostridium* (all *P* < 0.001), while the relative abundances of *Faecalibacterium, Phascolarctobacterium, Alistipes*, and *Sutterella* were significantly lower compared to the HC group (all *P* < 0.001). The observed increase in *Blautia* and *Lachnoclostridium*, both known to be acetate-producers (Vacca et al., 2020), might suggest a shift in short-chain fatty acid (SCFA) production profiles, although the net functional impact requires further investigation. Notably, the depletion of *Faecalibacterium*—a genus renowned for its anti-inflammatory properties, production of the SCFA butyrate, and role in maintaining intestinal barrier integrity (Lopez-Siles et al., 2017)—likely contributes to the low-grade systemic inflammation and disruption of the gut environment observed in GDD patients. The reduction of *Alistipes* and *Sutterella*, which have been implicated in tryptophan metabolism and mucosal immunity, may further disrupt gut-brain communication (Parker et al., 2020; Kaakoush, 2020). These findings partially align with and extend those reported by Huang et al. (2024), highlighting a conserved gut microbiota signature in neurodevelopmental disorders. Furthermore, accumulating evidence supports the notion that gut microbiota dysbiosis can promote the pathogenesis of GDD through multiple mechanisms, including increased intestinal permeability, activation of glial cells, amplification of inflammatory responses, and induction of oxidative stress (Huang et al., 2024; Munoz-Pinto et al., 2024).

### 4.3 Synergistic damage to neuro development caused by abnormal gut microbiota function

In this study, we employed functional prediction to generate hypotheses regarding the potential metabolic consequences of the observed microbiota dysbiosis. The analysis suggested that the gut microbiome of children with GDD might have an altered functional potential, particularly in three key pathways: 1) mRNA surveillance pathway: *Certain gut microbiota may activate localized mRNA translation through abnormal metabolites* (such as GABA) (Huang et al, 2022), which interferes with neuronal dendritic development and synaptic connection; 2) Proteasomal pathway: The decrease in butyric acid caused by the absence of faecal bacteria may weaken proteasomal function (Davidson and Pickering, 2023), exacerbate erroneous protein aggregation and neuroinflammation; 3) Atrazine degradation: Up-regulation of the Atrazine degradation pathway suggests that exposure to environmental toxin may accelerate neural stem cell senescence through microbial metabolites(Genovese et al., 2021; Chen et al., 2024), but the exact mechanism needs to be further verified in the context of toxin exposure history.

Compared with the HC, functional prediction based on 16S rRNA data suggested potential upregulation of gut microbiota functions in the GDD group, including mRNA surveillance pathway, Proteasome pathway, and Atrazine degradation pathway. Conversely, significantly downregulated functions included Polyketide sugar unit biosynthesis, Chloroalkane and chloroalkene degradation, and Styrene degradation (**Figure 4**). The downregulation of polyketide sugar unit biosynthesis may reflect a reduction in the production of secondary metabolites, which have been implicated in maintaining gut-brain homeostasis (Kim et al., 2024). Similarly, impaired degradation pathways for environmental toxins such as chloroalkanes and styrene could indicate a diminished capacity for detoxification, potentially exacerbating neurotoxic effects (Zhou et al., 2024). These functional shifts, while derived from predictive analyses, suggest a broader disruption in microbial metabolic support and environmental resilience in GDD.

The functional predictions (e.g., upregulation of mRNA surveillance and proteasome pathways) were derived from 16S rRNA data using PICRUSt2, which infers metagenomic potential rather than measuring actual gene expression or metabolite levels. Consequently, these findings represent hypotheses about functional consequences rather than direct evidence. The predicted upregulation in the atrazine degradation pathway, for instance, might indicate a microbial response to environmental exposures, but its direct impact on neural stem cells remains speculative without measuring serum or fecal metabolites. It should be noted that these functional predictions are inferred from 16S rRNA sequencing data using PICRUSt2 and have not been validated by metagenomic or metabolomic analyses. Therefore, they represent hypothetical functional shifts rather than confirmed metabolic activities.

### 4.4 Generalizability and regional considerations

As a single-center study conducted in Shenzhen, China, our findings should be interpreted with consideration of regional and institutional factors. The dietary habits, environmental exposures, and genetic backgrounds of the participating children may reflect the characteristics of an urban population in Southern China, which could limit the direct applicability of our results to other geographic or cultural settings. For instance, dietary patterns rich in certain fermented foods or exposure to local environmental toxins (e.g., pesticides) may influence gut microbiota composition. Future multi-center studies involving diverse populations are needed to validate these findings and enhance their generalizability.

## 5.Conclusion

This study provides the first systematic characterization of the gut microbiota in children with GDD, revealing distinct structural and functional alterations. Our findings suggest a significant reduction in microbial alpha diversity and notable shifts in community composition, including a marked decrease in beneficial butyrate-producing genera such as *Faecalibacterium* and an unexpected increase in *Bifidobacterium*. These microbial disturbances are potentially linked to neuroinflammatory pathways and oxidative stress, suggesting a plausible mechanism through which the gut microbiota may influence neurodevelopment. The observed gut microbiota profile partially overlaps with those reported in ASD and ADHD, supporting the concept of a shared gut-brain axis pathology across neurodevelopmental disorders. However, the cross-sectional design of this study limits causal inference regarding the observed associations. Future longitudinal studies with serial assessments are warranted to elucidate the temporal relationship between gut microbiota dynamics and neurodevelopmental trajectories in GDD.

## 6 Limitations and future perspectives

While this study provides the first detailed characterization of the gut microbiota in children with GDD, several limitations should be considered when interpreting the results, which also pave the way for future research. First, the single-center design and modest sample size (n=26 in the GDD group) may limit the statistical power and generalizability of our findings. Future multi-center studies with larger cohorts are necessary to validate our observations and explore potential subgroup differences (e.g., based on GDD severity. subtype or therapeutic intervention). Second, as an observational study, our findings demonstrate an association but cannot establish a causal relationship between the gut microbiota and GDD. The functional predictions are derived from 16S rRNA data and require validation through direct methods such as shotgun metagenomics, metabolomics, and transcriptomics to confirm the actual microbial gene expression and metabolite production (e.g., butyrate, GABA). Third, although we matched for several baseline characteristics, the potential influence of unmeasured confounders, such as detailed dietary records, medication history, and household environment, cannot be fully excluded. Furthermore, the cross-sectional design does not capture the longitudinal dynamics of the gut microbiota. Prospective cohort studies following infants at risk for GDD from birth are needed to determine whether gut dysbiosis is a predisposing factor or a consequence of the disorder, and to correlate microbial changes with neurodevelopmental trajectories. Finally, the specific neuroprotective or neurotoxic mechanisms of the identified bacterial genera (e.g., *Faecalibacterium, Bifidobacterium*) and their metabolites remain hypothetical. Interventional animal models and *in vitro* studies are essential to definitively elucidate the underlying mechanisms of the gut-brain axis in GDD.

## Data Availability

The datasets presented in this study can be found in online repositories. The names of the repository/repositories and accession number(s) can be found in the article/supplementary material.

## References

[B1] Borghi E. Xynomilakis O. Ottaviano E. Ceccarani C. Viganò I. Tognini P. . (2024). Gut microbiota profile in CDKL5 deficiency disorder patients. Sci. Rep. 14, 7376. doi: 10.1038/s41598-024-56989-0, PMID: 38548767 PMC10978852

[B2] Chen J. Dai X. Y. Malhi K. K. Xu X. W. Tang Y. X. Li X. W. . (2024). A new insight into the mechanism of atrazine-induced neurotoxicity: triggering neural stem cell senescence by activating the integrated stress response pathway. Res. (Wash D.C.) 7, 547. doi: 10.34133/research.0547, PMID: 39679284 PMC11638487

[B3] Cryan J. F. Dinan T. G. (2012). Mind-altering microorganisms: the impact of the gut microbiota on brain and behaviour. Nat. Rev. Neurosci. 13, 701–712. doi: 10.1038/nrn3346, PMID: 22968153

[B4] Davidson K. Pickering A. M. (2023). The proteasome: A key modulator of nervous system function, brain aging, and neurodegenerative disease. Front. Cell Dev. Biol. doi: 10.3389/fcell.2023.1124907, PMID: 37123415 PMC10133520

[B5] Fu Y. Wang X. Nie L. Wang Z. Ma X. Wu L. . (2025). Gut microbiota characteristics in neonatal respiratory distress syndrome and the therapeutic potential of probiotics in recovery. Front. Microbiol. 16. doi: 10.3389/fmicb.2025.1544055, PMID: 40256622 PMC12006762

[B6] Genovese T. Siracusa R. Fusco R. D'Amico R. Impellizzeri D. Peritore A. F. . (2021). Atrazine inhalation causes neuroinflammation, apoptosis and accelerating brain aging. Int. J. Mol. Sci. 22, 7938. doi: 10.3390/ijms22157938, PMID: 34360708 PMC8347547

[B7] Huang J. Jiang B. Li G.-W. Zheng D. Li M. Xie X. . (2022). m6A-modified lincRNA dubr is required for neuronal development by stabilizing YTHDF1/3 and facilitating mRNA translation. Cell Rep. 41, 111693. doi: 10.1016/j.celrep.2022.111693, PMID: 36417851

[B8] Huang M. Yu X. T. Zhou P. Lu Z. S. Zhu S. H. Nie Y. . (2024). Expression of insulin-like growth factor 1, characteristics of gut microbiota and their correlation in children with cerebral palsy and growth retardation. Chin. J. Sch. Health 1246–1250, 1254. doi: 10.16835/j.cnki.1000-9817.2024290

[B9] Kaakoush N. O. (2020). Sutterella species, igA-degrading bacteria in ulcerative colitis. Trends Microbiol. 28, 519–522. doi: 10.1016/j.tim.2020.02.018, PMID: 32544438

[B10] Kim N. Y. Lee H. Y. Choi Y. Y. Mo S. J. Jeon S. Ha J. H. . (2024). Effect of gut microbiota-derived metabolites and extracellular vesicles on neurodegenerative disease in a gut-brain axis chip. Nano. Converg. 11, 7. doi: 10.1186/s40580-024-00413-w, PMID: 38340254 PMC10858859

[B11] Lopez-Siles M. Duncan S. H. Garcia-Gil L. J. Martinez-Medina M. (2017). Faecalibacterium prausnitzii: from microbiology to diagnostics and prognostics. ISME. J. 11, 841–852. doi: 10.1038/ismej.2016.176, PMID: 28045459 PMC5364359

[B12] Lou M. Cao A. Jin C. Mi K. Xiong X. Zeng Z. . (2022). Deviated and early unsustainable stunted development of gut microbiota in children with autism spectrum disorder. Gut 71, 1588–1599. doi: 10.1136/gutjnl-2021-325115, PMID: 34930815 PMC9279844

[B13] Miclea D. Peca L. Cuzmici Z. Pop I. V. (2015). Genetic testing in patients with global developmental delay/intellectual disabilities. A review. Cluj. Med. 88, 288–292. doi: 10.15386/cjmed-461, PMID: 26609258 PMC4632884

[B14] Munoz-Pinto M. F. Candeias E. Melo-Marques I. Esteves A. R. Maranha A. Magalhães J. D. . (2024). Gut-first parkinson’s disease is encoded by gut dysbiome. Mol. Neurodegener. 19, 78. doi: 10.1186/s13024-024-00766-0, PMID: 39449004 PMC11515425

[B15] National Center for Children's Health, Healthcare Center of Beijing Children's Hospital Affiliated to Capital Medical University, Beijing Pediatric Quality Control and Improvement Center, Professional Committee of Child Brain Science and Brain Health Promotion of China Maternal and Child Health Association, Professional Committee of Child and Adolescent Health Development of Beijing Health Culture Promotion Association, Professional Committee of Autism Prevention and Treatment Research of Maternal Child Health Research Association, Developmental Behavioral Pediatrics Group of Chinese Society of Pediatrics . (2024). Chinese guideline for the diagnosis of global developmental delay. Chin. J. Pract. Pediatr. doi: 10.3760/cma.j.cn101070-20240426-00262

[B16] Nistal E. Caminero A. Herrán A. R. Arias L. Vivas S. de Morales J. M. R. . (2012). Differences of small intestinal bacteria populations in adults and children with/without celiac disease: effect of age, gluten diet, and disease. Inflamm. Bwl. Dis. 18, 649–656. doi: 10.1002/ibd.21830, PMID: 21826768

[B17] Parker B. J. Wearsch P. A. Veloo A. C. M. Rodriguez-Palacios A. (2020). The genus alistipes: gut bacteria with emerging implications to inflammation, cancer, and mental health. Front. Immunol. 11. doi: 10.3389/fimmu.2020.00906, PMID: 32582143 PMC7296073

[B18] Peng Y. Chiu A. T. G. Li V. W. Y. Zhang X. Yeung W. L. Chan S. H. S. . (2023). The role of the gut-microbiome-brain axis in metabolic remodeling amongst children with cerebral palsy and epilepsy. Front. Neurol. 14. doi: 10.3389/fneur.2023.1109469, PMID: 36923492 PMC10009533

[B19] Song Q. Wang J. Wei C. L. Cao F. Z. Zhang L. J. (2024). Longitudinal study of magnetic resonance diffusion tensor imaging in prognostic evaluation of children with intellectual disability/global developmental delay. J. Imaging Res. Med. Appl. 74–76, 80. doi: 10.3969/j.issn.2096-3807.2024.24.022

[B20] Tognini P. (2017). Gut microbiota: a potential regulator of neurodevelopment. Front. Cell. Neurosci. 11. doi: 10.3389/fncel.2017.00025, PMID: 28223922 PMC5293830

[B21] Vacca M. Celano G. Calabrese F. M. Portincasa P. Gobbetti M. De Angelis M. (2020). The controversial role of human gut lachnospiraceae. Microorganisms 8, 573. doi: 10.3390/microorganisms8040573, PMID: 32326636 PMC7232163

[B22] Vickers R. R. Gibson J. S. (2019). A review of the genomic analysis of children presenting with developmental delay/intellectual disability and associated dysmorphic features. Cureus 11, e3873. doi: 10.7759/cureus.3873, PMID: 30899624 PMC6420327

[B23] Volpedo G. Riva A. Nobili L. Zara F. Ravizza T. Striano P. (2025). Gut-immune-brain interactions during neurodevelopment: from a brain-centric to a multisystem perspective. BMC Med. 23, 263. doi: 10.1186/s12916-025-04093-z, PMID: 40325407 PMC12054192

[B24] Wan L. Ge W. R. Zhang S. Sun Y. L. Wang B. Yang G. (2020). Case-control study of the effects of gut microbiota composition on neurotransmitter metabolic pathways in children with attention deficit hyperactivity disorder. Front. Neurosci. 14, 127. doi: 10.3389/fnins.2020.00127, PMID: 32132899 PMC7040164

[B25] Wang X. Wang N. Gao T. Zhang Y. Fu Z. Zhao Y. . (2025). Symptom-specific gut microbial and metabolic profiles in ADHD reveal SCFA deficiency as a key pathogenic mechanism. Gut. Microbes 17, 2537755. doi: 10.1080/19490976.2025.2537755, PMID: 40719366 PMC12309550

[B26] Ye F. Gao X. Wang Z. Cao S. Liang G. He D. . (2021). Comparison of gut microbiota in autism spectrum disorders and neurotypical boys in China: a case control study. Synth. Syst. Biotechnol. 6, 120–126. doi: 10.1016/j.synbio.2021.03.003, PMID: 34095558 PMC8163862

[B27] Zhang L. Xu Y. Li H. Li B. Duan G. Zhu C. (2022). The role of probiotics in children with autism spectrum disorders: a study protocol for a randomised controlled trial. PLoS One 17, e0263109. doi: 10.1371/journal.pone.0263109, PMID: 35202432 PMC8870536

[B28] Zhou Y. Zhang L. Li Q. Wang P. Wang H. Shi H. . (2024). Prenatal PFAS exposure, gut microbiota dysbiosis, and neurobehavioral development in childhood. J. Haz. Mater. 469, 133920. doi: 10.1016/j.jhazmat.2024.133920, PMID: 38457972

